# Visualization toolkits for enriching meta-analyses through evidence maps, bibliometrics, and alternative impact metrics

**DOI:** 10.1017/rsm.2024.3

**Published:** 2025-03-19

**Authors:** Yefeng Yang, Malgorzata Lagisz, Shinichi Nakagawa

**Affiliations:** 1 Evolution & Ecology Research Centre and School of Biological, Earth and Environmental Sciences, University of New South Wales, Sydney, NSW, Australia; 2 Department of Biological Sciences, University of Alberta, Edmonton, AB, Canada

**Keywords:** data visualization, evidence and gap maps, evidence synthesis, information science, large language models, scientometrics

## Abstract

Data visualization is crucial for effectively communicating knowledge in meta-analysis. However, existing visualization methods in meta-analysis have predominantly focused on quantitative aspects, such as forest plots and funnel plots, thereby neglecting qualitative information that is equally important for end-users in science, policy, and practice. We introduce a framework consisting of a series of visualization toolkits designed to enrich meta-analyses by borrowing approaches from other research synthesis methods, including systematic evidence mapping (scoping reviews), bibliometrics (bibliometric analysis), and alternative impact metric analysis. These “enrichment” toolkits aim to facilitate the synthesis of both quantitative and qualitative evidence, along with the assessment of the academic and nonacademic influences of the meta-analytic evidence base. While the meta-analysis yields quantitative insights, the enrichment analyses, and visualizations provide user-friendly summaries of qualitative information on the evidence base. For example, a systematic evidence map can visualize study characteristics, unraveling knowledge gaps and methodological differences. Bibliometric analysis offers a visual assessment of the nonindependent evidence, such as hyper-dominant authors and countries, and funding sources, potentially informing the risk of bias. Alternative impact metric analysis employs alternative metrics to gauge societal influence and research translation (e.g., policy and patent citations) of studies in the meta-analysis. We provide a dedicated webpage showcasing sample visualizations and providing step-by-step implementation in open-source software R (https://yefeng0920.github.io/MA_Map_Bib/). Additionally, we offer a guide on leveraging three commercially free large language models (LLMs) to help adapt the sample script, enabling users with less R coding experience to visualize their own meta-analytic evidence base.

## Highlights

### What is already known

- Data visualization enhances meta-analysis, aiding in identifying patterns and increasing the accessibility of evidence to end-users in science, policy, and practice.
- Quantitative information in meta-analyses is commonly presented through user-friendly formats, such as forest plots and funnel plots, with effect size estimates and precisions.
- Meta-analyses include qualitative information on study characteristics and bibliographic attributes, which are rarely visualized.

### What is new

- Integrating the elements of scoping reviews and bibliometrics into meta-analyses enriches research synthesis by visualizing qualitative evidence.
- Scoping reviews (systematic mapping) can enhance meta-analyses by providing a user-friendly summary of study characteristics and identifying knowledge gaps and gluts.
- Bibliometric analysis can visualize nonindependent evidence, revealing dominant authors and countries, informing potential risk of bias (RoB).
- Further, alternative impact metrics, such as Altmetric attention scores, patents, and policy citations, can help capture the societal impacts of meta-analyses beyond academia.

### Potential impact for RSM readers

- The proposed visualization toolkits transform qualitative information into easily digestible formats for end-users, requiring minimal additional data extraction effort from meta-analysts.
- The proposed visualization toolkits’ capacity can be extended to other methods in research synthesis ecosystems, such as the systematic-review family, review-of-review family, and rapid-review family, to convey quantitative information in a user-friendly manner for every evidence base.

## 1 Introduction

Data visualization is an effective tool for knowledge communication. It also works in meta-analysis,^1^ ^–^ ^3^ a statistical method for quantitatively synthesizing evidence.^4^ ^–^ ^12^ The incorporation of data visualization can yield two potential benefits to meta-analysis. First, data visualization can facilitate the identification of patterns, trends, and associations, not immediately apparent within the complex meta-analytic data.^13^ Second, it could increase the uptake and interpretation of meta-analytic findings by the end-users in science, policy, and practice.^2^ ^,^ ^14^ ^,^ ^15^

Typical meta-analytic data include both quantitative and qualitative information. Quantitative information usually refers to the effect size estimates, sampling variances, and quantitative moderators. Qualitative information comes in two types: study characteristics (e.g., contextual factors of the performed research) and bibliographic attributes (e.g., author and country information). Conventionally, the meta-analytic visualization methods have primarily focused on the quantitative aspects,^1^ ^,^ ^13^ exemplified by forest plots and funnel plots for representing effect sizes and their precisions.^3^ However, qualitative information is rarely visualized or tends to be conveyed in a format not immediately digestible by end-users.^16^ Comprehensive and clear visual representation of moderator variables can facilitate customizable evidence synthesis, identifying context-specific drivers and delivering more tailored evidence for science, policy, and practice, improving the generalizability and transferability of evidence.^17^ Yet, these qualitative details are no less valuable than their quantitative counterparts.

A user-friendly summary of study characteristics can add value to meta-analyses. For example, visualizing the intersections between the types of interventions and outcomes (or other combinations of two categorical variables) can identify knowledge gaps,^18^ thus setting the agenda for future research to fill such gaps. At the same time, identifying evidence “gluts” is of urgent importance given research waste due to preventable problems,^19^ such as designing new experiments while ignoring the existing evidence.^20^ The need for such visualizations aligns with the tenets of scoping reviews, sometimes termed evidence review or map (see ^21^ ^,^ ^22^ for a debate).^23^ ^,^ ^24^ Interestingly, network meta-analyses offer a precursor to integrating evidence mapping into meta-analyses. The geometry of network graphs can visually unveil the state of the evidence, distinguishing well-studied interventions from those warranting further investigation.^25^

Similarly, visualizing bibliographic attributes remains relatively rare in meta-analyses. Yet, such visualizations can reveal the nonindependence of evidence.^26^ For example, bibliometric analyses can map author collaboration networks,^27^ define research groups, and detect hyper-dominant author clusters (see ^28^ for an example).^29^ Such hyper-dominance induces nonindependent evidence and could influence between-study level risk of bias (RoB). Bibliometric analysis of traditional impact metrics (e.g., citations) can be used to map the conceptual influences within academia. Additionally, alternative impact metrics^30^ ^–^ ^33^ have been proposed to complement traditional citation impact metrics. These alternative impact metrics, such as Altmetric attention scores, can reflect the social impact of a paper based on online news media (e.g., Facebook), policy documents, patents, Wikipedia, and Stack Overflow, reflecting the influences outside academia.

**Figure 1:**
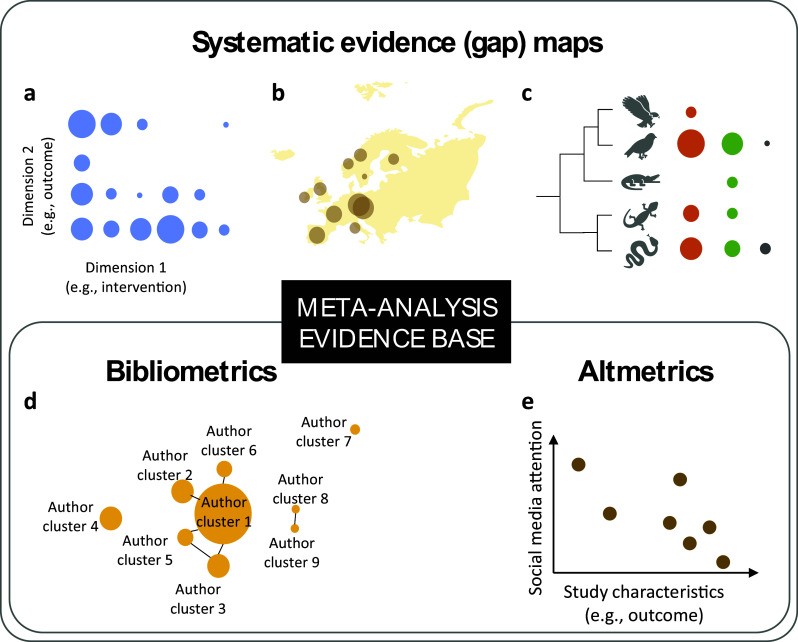
A conceptual diagram of the visualization framework illustrating the use of the proposed visualization approaches to enrich meta-analyses. These “enrichment” visualization approaches are adapted from research synthesis methods in the research synthesis method ecosystem, including systematic evidence map, bibliometrics (bibliometric analysis), and alternative impact metric analysis. Presented are selected hypothetical examples for the types of plots discussed in the main text. Panels (a) (grid-like graph), (b) (geographical map), and (c) (phylogenetic tree) are examples of plots used in systematic evidence maps. Panel (d) (co-authorship network) is an example of a plot used in bibliometrics. Panel (e) is a visual representation of the relationship between social media attention (e.g., Altmetric score) and study characteristics in a meta-analysis evidence base. For real examples, refer to Sections 3–5. A step-by-step implementation guide is available at https://yefeng0920.github.io/MA_Map_Bib/.

In this work, we present a series of visualization toolkits that enrich traditional meta-analyses by seamlessly integrating visualizations used in three research synthesis approaches: evidence mapping, bibliometrics, and alternative impact analyses (Figure 1). We explain the rationale of each “enrichment” approach and corresponding visualization toolkits. We then present a dedicated webpage with online step-by-step implementations of all these toolkits in R (https://yefeng0920.github.io/MA_Map_Bib/). For users with less R coding experience, we offer a guide on using three commercially free large language models (ChatGPT [https://openai.com/chatgpt/], Gemini [https://gemini.google.com/], and Copilot [https://copilot.microsoft.com/]) to modify the sample R script easily. Also, we use examples from diverse disciplines to illustrate the principles and procedures. The elegance of these proposed visualization toolkits lies in converting the qualitative information into readily digestible formats for end-users with minimal additional data extraction effort from meta-analysts. The visualization toolkits can also enrich other research synthesis methods in the research synthesis method ecosystem, including the systematic-review family (e.g., systematic reviews without meta-analysis), review-of-review family (e.g., overview, umbrella review), and rapid-review family (e.g., rapid review).^34^ ^,^ ^35^

## 2 Visualization toolkits for integrating evidence maps, bibliometrics, and altmetrics into meta-analysis

The three enrichment analyses of qualitative information (details see below) can be implemented in conjunction with a broad spectrum of meta-analytic approaches used across different fields, including conventional random-effects, multilevel, multivariate, dose–response, longitudinal, network, location-scale, individual participant data (IPD), phylogenetic comparative models, and meta-meta-analysis.^4^ ^,^ ^11^ Below, we present the details and case studies of the three enrichment approaches.

## 3 Evidence map in meta-analytic evidence base

### 3.1 Rationale

Evidence maps (scoping reviews) represent a form of research synthesis approach that systematically collates and catalogs existing evidence on a specific topic, yet is broader than a systematic review.^36^ While similar approaches exist with varying terminologies, such as mapping reviews, systematic evidence maps, and evidence and gap maps,^21^ ^–^ ^23^ ^,^ ^25^ ^,^ ^37^ ^,^ ^38^ here we treat them as synonyms because they share a common core feature: mapping the state of the evidence, which makes them valuable for meta-analysis enrichment. We use the term “evidence mapping,” defining it as a process that employs various visualization methods to provide a user-friendly summary of study characteristics (qualitative evidence) within a meta-analysis (the end-product).^16^ ^,^ ^22^ Incorporating evidence mapping into a meta-analysis offers two benefits. First, it inherits the merits of a conventional evidence map, such as identifying knowledge gaps, directing research priorities, and providing support for funding and policy decisions.^16^ ^,^ ^25^ Second, evidence mapping can inform methodological decisions (e.g., *post hoc* analyses) and interpretations of meta-analytic findings. Various visualization approaches exist, such as heatmap and bubble plots^39^ and other displays used in the 3ie Evidence Gap Map (https://www.3ieimpact.org/evidence-hub/evidence-gap-maps)^16^ and Evidence-based Policing Matrix (https://cebcp.org/evidence-based-policing/the-matrix/),^40^ for mapping the evidence landscape.

We recommend using grid-like graphs, where one dimension (e.g., intervention) is placed on the *x*-axis, and the other (e.g., outcomes) is placed on the *y*-axis, with statistics (e.g., the number of studies, number of effects, sample size) displayed at the intersection of the *x*- and *y*-axes.^18^ Additional dimensions, such as study design, effect size, study quality, or RoB measures, can also be incorporated (see more on this point in the next section). We recommend using alluvial or Sankey diagrams to visualize the flow or overlaps in the composition of context-dependence drivers, summarizing their connections and co-linearity, and missing data patterns in an accessible manner. Additionally, a geographical map of study participants could reveal the representation status of historically underrepresented regions or groups, by incorporating location, gender, ethnic, or social backgrounds into visualizations. Understanding such patterns, in turn, can facilitate actions toward greater equity, diversity, and inclusion (EDI) in research. For phylogenetic meta-analytic analyses, visual representation of species information, such as species phylogenetic trees, can intuitively convey the breadth of taxa and underlying phylogenetic heterogeneity.

### 3.2 Case studies

We demonstrate the application of evidence mapping using three publicly available meta-analytic datasets (Data 1 to Data 3) from various disciplines.^41^ ^–^ ^43^ Note that these applications are provided for illustrative purposes, and any scientific, clinical, or policy implications should not be drawn from them. The data and code for replicating all visualizations can be found at https://yefeng0920.github.io/MA_Map_Bib/.

Data 1: Hodkinson et al.^41^ conducted a network meta-analysis (including 68 studies and 80 effect size estimates) to assess the efficacy of different self-management interventions (multidisciplinary case management, regularly supported self-management, and minimally supported self-management) in enhancing the quality of life among asthma patients.

Data 2: Mertens et al.^42^ employed a multilevel meta-analytic model to synthesize evidence (including 212 studies and 447 effect size estimates) on the effectiveness of choice architecture interventions (often referred to as nudges) for behavior change across various techniques, behavioral domains, and other study characteristics (e.g., populations and locations).

Data 3: Sanders et al.^43^ used a Bayesian meta-analytic model to synthesize evidence (including 126 studies and 1,304 effect size estimates) regarding the impacts of artificial light at night on physiological, phenological, life history, activity patterns, and population/community-based outcomes. This meta-analysis included more than 180 species. For illustration, we used the subset that focused on physiological outcomes.

Evidence maps (scoping reviews) often use structured formats to map the evidence of a given field, such as participant, context, concept (PCC) and units, treatments, outcomes, or settings (UTOS).^18^ ^,^ ^21^ For Data 1, we used grid-like graphs with the intervention variable on the *x*-axis and the outcome variable on the *y*-axis.^18^ This aimed to identify knowledge gaps in self-management interventions. Each cell displayed two statistics: the number of studies and the number of effect sizes. We further quantified the population mean effect size estimate for each cell using a robust point and variance estimation approach.^44^ For Data 2, we utilized an alluvial diagram to illustrate the connections (collinearities) between moderator variables related to the hypotheses tested. Data 3 featured a phylogenetic tree to visualize the taxonomic breadth and relatedness among the species involved. Data processing and visualizations were conducted using packages *ggplot2*,^45^ *ape*,^46^ *ggtree*,^47^ and *metafor.* ^48^

### 3.3 Results and discussion

**Figure 2:**
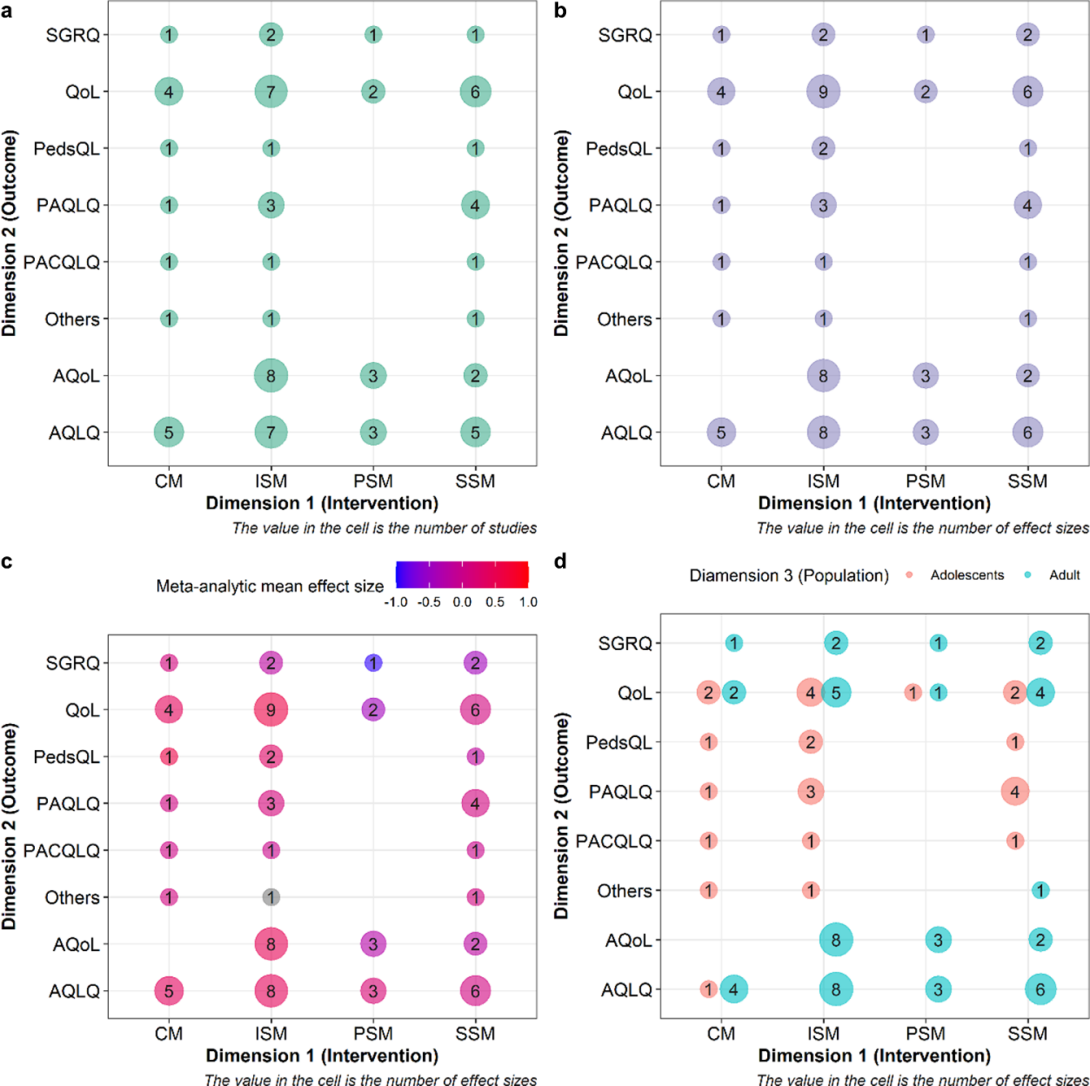
Examples of evidence maps visualizing study characteristics. (a) A typical grid-like graph with the intervention variable as the first dimension, the outcome variable as the second dimension, and the bubble size representing the number of studies. (b) The bubble sizes are changed to represent the number of effect sizes. (c) The color scale is applied to the bubbles to denote the magnitude of the overall mean effect size. Note that the application of Figure 2 requires that outcomes and interventions (or any other two variables reflecting study characteristics) could be categorized into different levels via moderators, as shown in Figure 2. Hedge’s g was used as the effect size measure to quantify mean differences in outcomes between an intervention and a control group. A multivariate fixed effect model was used to derive the overall average effect of each paired combination of intervention and outcome type.^44^ (d) The population variable is mapped to the shape serving as the third information dimension. The full names of different types of interventions are as follows: CM = case management, ISM = intensive self-management, PSM = pure self-management, SSM = support self-management. The full names of different types of outcomes are as follows: SGRQ = St Georges Respiratory Questionnaire, QoL = Quality of Life Scale, PedsQL = Pediatric Quality of Life Inventory Generic Core Scales, PAQLQ = Pediatric Asthma Quality of Life Questionnaire, PACQLQ = Pediatric Asthma Caregiver’s Quality of Life Questionnaire, AQoL = Assessment of Quality of Life, and AQLQ = Asthma Quality of Life Questionnaire. For the details of the interventions and outcomes, refer to the original paper.^41^ R code was adapted from Polanin et al.^18^

Figure 2 shows the landscape of evidence on the effectiveness of the self-management interventions in Data 1. For example, panels (a) and (b) visually represent where randomized controlled trials (RCTs) are available to examine the interventions’ effectiveness, offering insights into which interventions have received more clinical attention. Panel (c) conveys critical information about both which interventions have been examined in the RCTs and their associated effectiveness. Panel (d) reveals a potential demographic bias, as most self-management interventions were trialed on adults. This information can inform funding strategies (e.g., funding missing RCTs, or in-depth investigation) and help clinicians gauge the volume and effectiveness of their interventions of interest.

**Figure 3:**
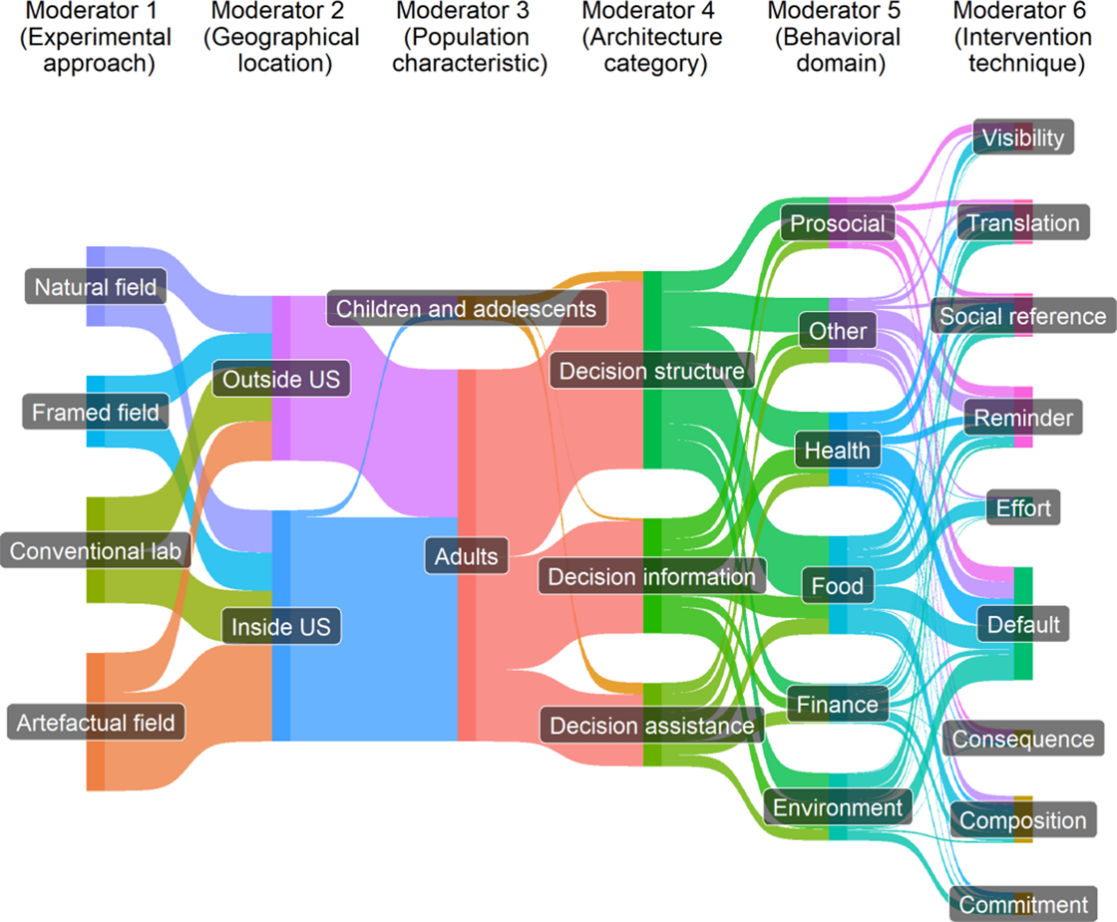
An example of an alluvial diagram showing the overlaps in the composition of moderator variables considered as potential context-dependence drivers of effect sizes in a meta-analysis.

Figure 3 highlights the diversity of experimental designs in the primary studies included, suggesting potential heterogeneity in the meta-analytic evidence in Data 2. The tested moderator variables display minimal collinearity, indicating that each variable represents a unique contextual influence. Importantly, Figure 3 provides useful visual clues to identify the contexts requested by decision-makers, facilitating the assessment of the effectiveness of interventions in the context of interest (e.g., target population and location). A follow-up customizable evidence synthesis can be conducted to improve the generalizability and transferability of meta-analytic evidence (see ^17^ for more details).

**Figure 4:**
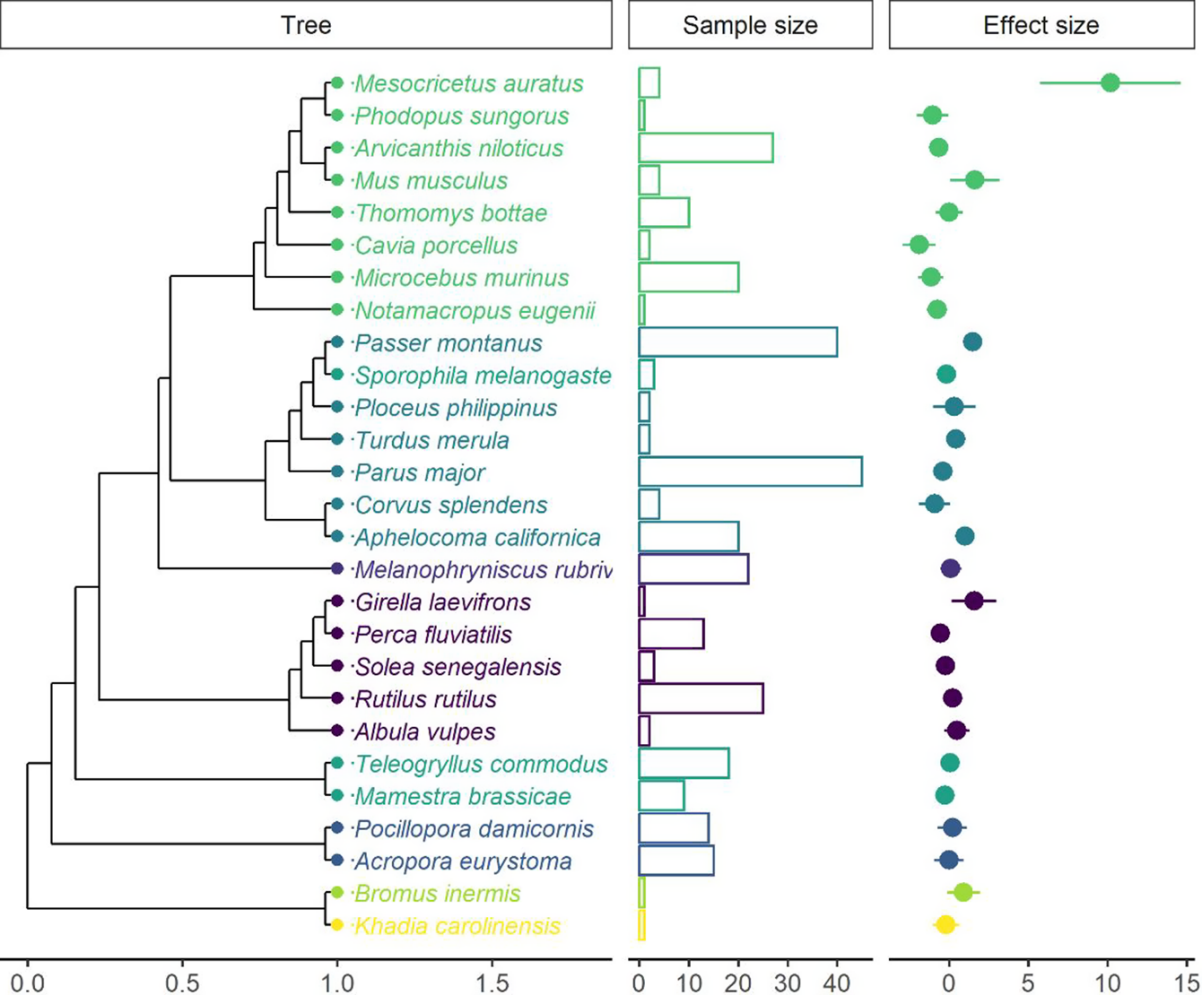
An example of a phylogenetic tree visualizing the breadth of taxa and underlying phylogenetic heterogeneity. The effect size estimates for each species were weighted based on the inverse of the sampling variance–covariance matrix. Confidence intervals for each species were constructed using a Ward-type approach, with standard errors as the square root of the sampling variance of the weighted mean. For simplicity, we assumed a sampling correlation of 0.5 for the calculation of confidence intervals (the actual correlation should be informed from ancillary data or guesstimates by domain experts). The dot represents the average mean effect size of each species. The whisker represents the 95% confidence interval. Different colors represent different phylogenetic classes. We approximated the branch lengths of the phylogenetic tree, using the Grafen method, setting the power parameter of 1 to adjust the “height” of branch lengths at the tips of the phylogenetic tree. The correlation matrix of the phylogenetic tree was estimated, assuming that the evolution of the trait follows the Brownian motion.

Figure 4 presents a typical phylogenetic tree revealing the broad coverage of taxa used in artificial light at night experiments, including birds, mammals, insects, reptiles, and arachnids in Data 3. For a more in-depth statistical analysis, constructing a phylogenetic correlation matrix can quantify the effect of the shared evolutionary history among species in the meta-analytic evidence base.

## 4 Bibliometric map in meta-analytic evidence base

### 4.1 Rationale

Bibliometric analysis, often called bibliometrics, is a technique that effectively synthesizes bibliometric data to provide an overview of the performance and intellectual structure of a field.^49^ It holds a unique position in the research synthesis method ecosystem because it synthesizes the bibliometric characteristics (literature metadata, not research content),^26^ such as authors, countries, funders, and citation counts.^50^ Literature-level characteristics are to scientific papers as epidemiology is to patients.^51^ Diagnosing the “epidemiological susceptibility risk” of studies can uncover hidden biases across the evidence base. Such a process can also extend the scope of RoB assessment from within-study to between-study levels (note that between-study RoB is usually known as publication bias^52^ while within-study RoB is just “risk of bias”). Studies are often not independent in terms of their conduct, with the same researchers potentially involved in multiple studies. Thus, highly represented authors (or research groups) may potentially dominate the production of evidence, limiting methodological diversity and generalizability. Indeed, centralized collaboration communities, which involve shared authors and employ similar protocols, have been shown to yield less replicable findings than decentralized ones, which incorporate more diverse independent research groups.^53^ Moreover, the publication of false, exaggerated, and falsified effects is believed to be more common in countries with a “publish or perish” culture.^54^ ^,^ ^55^ For example, meta-scientific evidence suggests that studies from the United States tend to overestimate effect sizes and exhibit a larger publication bias.^56^ ^–^ ^58^

Bibliometrics can bring two major additions to meta-analyses. Bibliometrics can construct a co-authorship network for the studies included in a meta-analysis. Mapping the co-authorship network plot can intuitively reveal authorship dependence.^29^ Network metrics, such as the degree of centrality, can provide quantitative insights. Bibliometrics can also identify dominant and unrepresented countries of author affiliation, or reveal interdependences between countries. Following similar principles, bibliometrics can identify research location bias (e.g., studies from high-impact or predatory journals),^59^ ^,^ ^60^ funding source bias,^61^ ^,^ ^62^ linguistic bias,^63^ ^,^ ^64^ and time-lag bias.^65^ ^,^ ^66^ Notably, a quantitative assessment of those factors can be accomplished by conducting subgroup analysis or meta-regression.^4^

### 4.2 Case studies

We use Data 3^43^ to illustrate the application of bibliometric analysis in meta-analytic evidence base. We collected bibliographic data for the primary studies included in the meta-analysis from Scopus using digital object identifier) searches. We created two bibliographic network typologies using the associated data: co-authorship and country of affiliation. The co-authorship network was constructed through collaboration analysis by multiplying two matrices, *Author* × *Paper* and *Paper* × *Author*. For the country network, bibliographic coupling was used, involving multiplication of *Paper* × *Cited paper* and *Cited paper* × *Paper*. We used *bibliometrix* package^27^ to construct network. Packages *igraph* ^67^ and *circlize* ^68^ were used to project the networks.

### 4.3 Results and discussion

**Figure 5:**
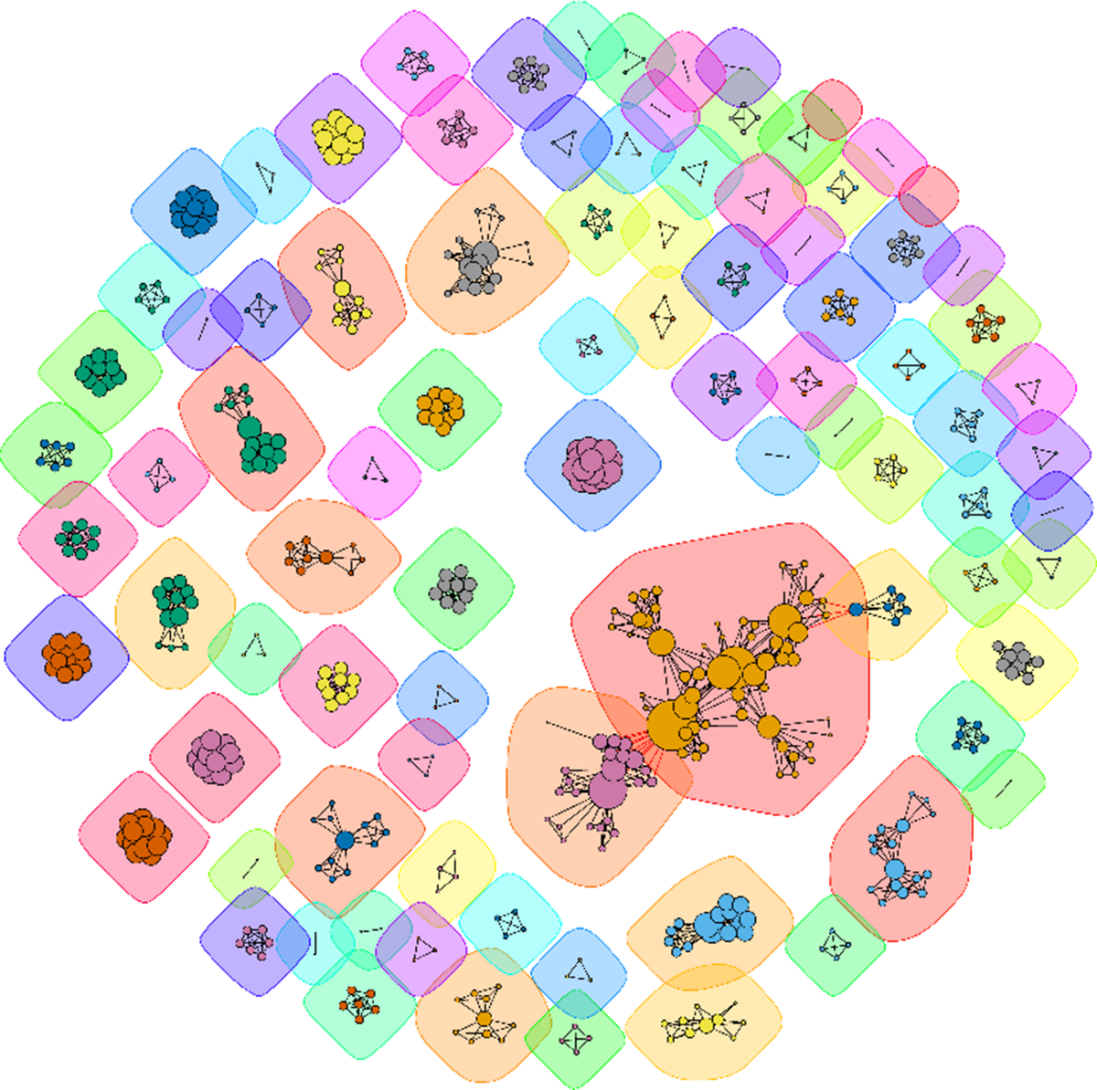
An example of a co-authorship network visualizing the diversity of the research groups and the degree of centralization of the scientific community. The vertices (nodes) and edges (links) denote authors and co-authorships, respectively. Bubbles of the same color represent the same author cluster or research team. Each color denotes a co-authorship cluster (or research group). The figure was inspired by Moulin et al.,^29^ who originally implemented it using Matlab and VOSviewer.

Figure 5 visually depicts the co-author network, with vertices (circles) representing authors and edges (lines) representing co-authorships in Data 3. Highly represented authors and dominant research groups can produce the majority of the evidence, reducing its methodological diversity and generalizability (certain groups might report certain results that are caused by bias in experimental designs and analyses).^29^

**Figure 6:**
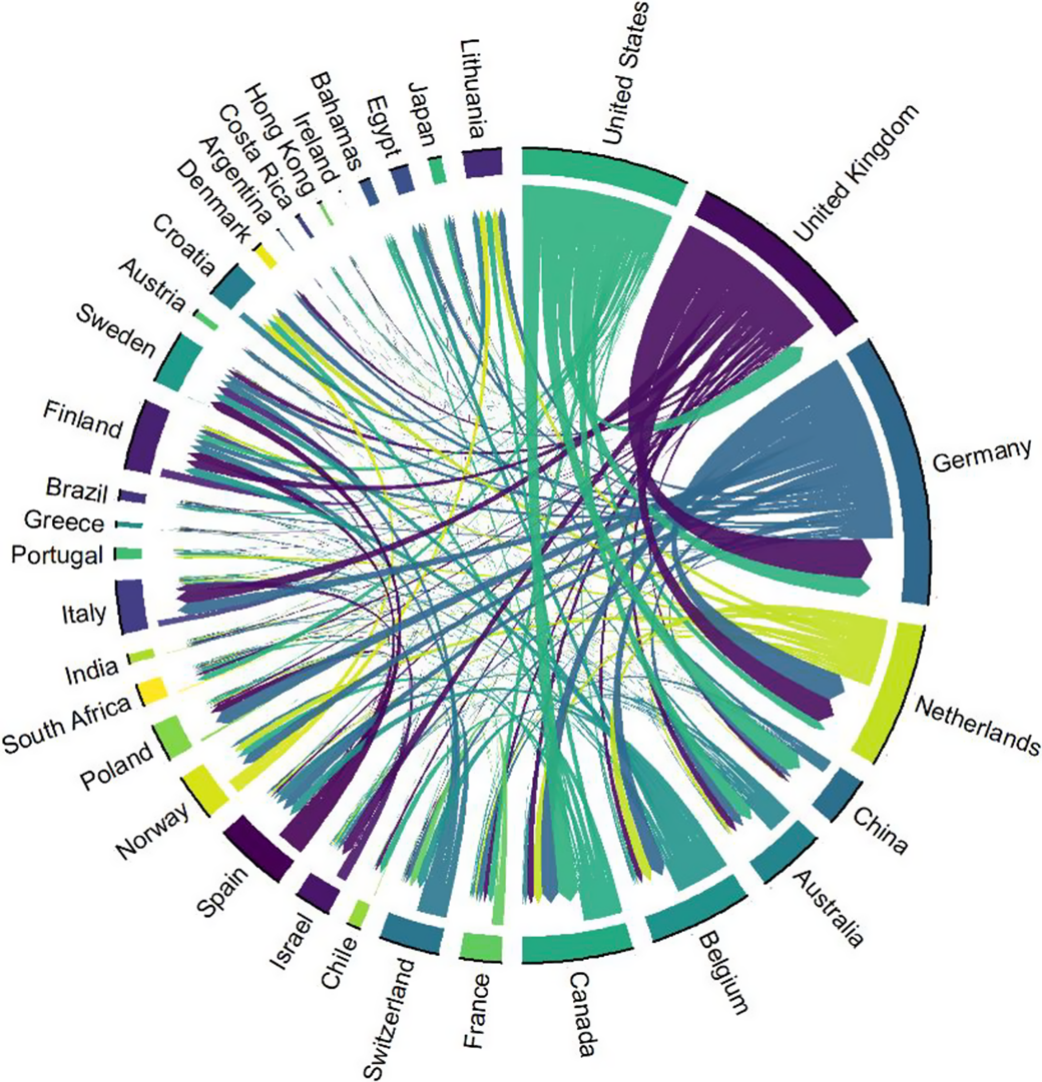
An example of a chord diagram showing the epistemological interdependences between different countries of author affiliation in the meta-analytic evidence base. These inter-dependences are quantified using a bibliographic coupling approach. Two countries are coupled when the cumulative bibliographies of their respective papers share one or more cited references. The coupling strength, an indicator of dominance, increases as the number of co-cited references between them increases.

In the example meta-analytic data set, we observe nearly 90 author clusters, each of which, on average, included six authors. The largest cluster had 58 authors. To quantitatively detect the effect of hyper-dominant research groups on meta-analytic estimates, a leave-one-out analysis can be employed^4^ to compare the meta-analytic effect size estimates of each research group with that of the rest. Figure 6 shows the country of affiliation citation network with 34 countries of author affiliation. While there is no obvious indication of dominant countries (with country contributions shown as proportion of the circle’s perimeter), the United States, the United Kingdom, and Germany are the most prominent players in this field, and all countries appear to be linked well via article citations. At the statistical follow-up work, a multilevel meta-analytic model with random effects at the levels of author and country of affiliation clusters can be used to correct potential biases in country influence.^4^ ^,^ ^29^

## 5 Alternative metric analysis of meta-analytic evidence bases

### 5.1 Rationale

Analysis of alternative impact metrics, termed “Altmetrics,” is an approach used to estimate research impact beyond academia.^33^ It can quantify societal impact by tracking activities on social media (e.g., Facebook and X [formerly Twitter]), Internet pages (e.g., Wikipedia), policy-related documents, and patent applications.^33^ ^,^ ^69^ ^–^ ^71^ Incorporating Altmetrics into a meta-analysis can reveal the uptake of research findings by stakeholders outside academia. For example, funding agencies have explored utilizing Altmetrics to assess public engagement with the research they support.^72^ ^–^ ^74^ Similarly, policymakers have used it to determine early engagement with research within the policy domain.^75^ We recommend visualizing the overall scores (e.g., Altmetric attention score), policy citations, patent citations, and overall social media attention received by studies within a meta-analysis to identify the extent to which these studies are translated into policy, practical applications, or general knowledge dissemination, respectively.

### 5.2 Case studies

We use one meta-analytic data to examine the replicability of the preclinical cancer biology.^76^ to illustrate the application of Altmetrics analysis, based on data from Altmetrics online service (www.altemetric.com) (Data 4). All data and code necessary for reproducing the subsequent visualizations can be accessed at the https://yefeng0920.github.io/MA_Map_Bib/. The data used for this demonstration are derived from the work of Errington et al.,^76^ who conducted a meta-analysis on the differences between the original effect size estimates and replicated effect size estimates across 50 cancer biology experiments. These experiments were sourced from 23 papers published in high-profile journals, such as *Nature*, *Science*, *Cell*, and *PNAS*.

We obtained Altmetric scores for each paper included in the meta-analytic dataset using the Altmetrics online service. This online service also provides data on the counts of policy documents and patent applications directly mentioned by organizational websites. We used API (application programming interface) to automatically retrieve the total Altmetric score, policy, and patent citation for each original publication. We visually represented the results using the orchard plot^77^ and grid-like graph.^18^

### 5.3 Results and discussion

**Figure 7:**
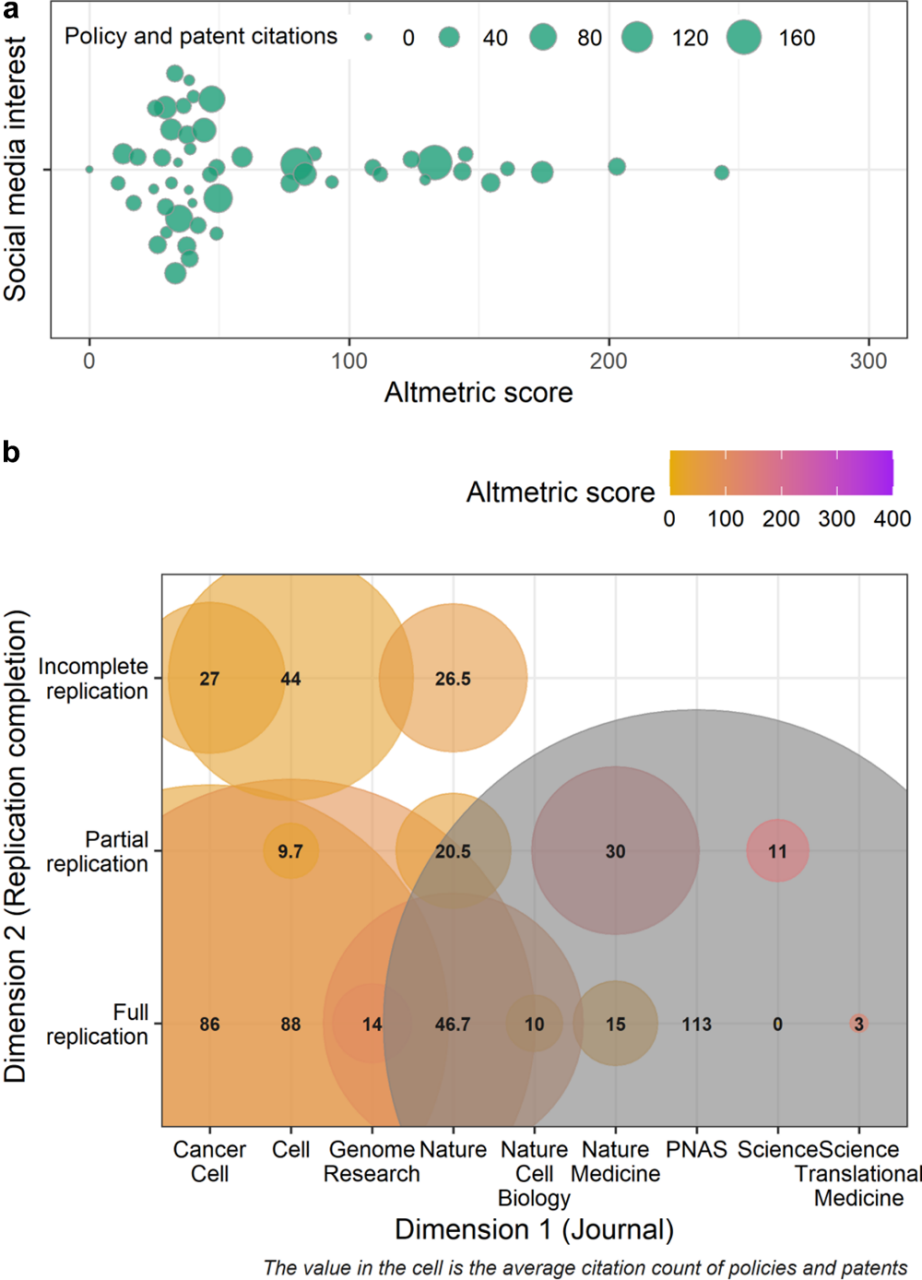
Examples of visualization showing the results of impact metric analysis. (a) An orchard plot with Altmetric score as the bubble and impact metric related to indicators of practical application (i.e., patent and policy citation counts) as the bubble size. (b) A grid-like graph where: 1) the color and size of the bubbles correspond to the Altmetric score, and 2) the impact metric counts, related to the indicator of practical translation (i.e., patent and policy citation counts), respectively. The grey bubble indicates that the Altmetric score exceeds 400. The categories of “full,” “partial,” and “no replication” denote that replication of studies was as fully replicated, partially replicated, or not replicated, respectively.

Figure 7a indicates the substantial social media attention earned by cancer biology studies included in the meta-analytic evidence base. Many of these studies were mentioned in policy documents and patent applications, indicating a potential degree of practical translation. Notably, fully replicated studies exhibited relatively higher Altmetric scores, and larger policy and patent citation counts compared to that of studies that were only partially replicated and not replicated. Among the fully replicated studies, those published in *PNAS*, *Cancer Biology*, and *Cell*, exhibited higher impact metrics than those published in *Nature* and *Science*.

## 6 Conclusions

Meta-analysis has become a widely accepted synthesis method across various disciplines. Meanwhile, scoping reviews (evidence maps), bibliometrics, and altmetrics are gaining popularity. We leverage the visualization approaches used in these three research synthesis methods to synthesize both quantitative and qualitative evidence. For meta-analysts, the proposed toolkits require minimal effort to locate, screen, code, and appraise additional evidence, as these procedures are typically integral to a standard meta-analysis. For end-users (e.g., researchers, policymakers, and practitioners), they summarize the qualitative part of the meta-analytic evidence base in immediately digestible graphs enriched with additional dimensions. These graphs can better visualize the landscape of the meta-analytic evidence base, facilitate the identification of knowledge gaps and methodological decisions, uncover hidden biases, and map the societal impact and degree of research translation. To facilitate the adoption of the proposed visualization toolkits, we have created a dedicated website offering step-by-step implementation guides using open-source software R (https://yefeng0920.github.io/MA_Map_Bib/). Given the dynamic nature of data visualization methods and R packages, we are committed to regularly updating our website to incorporate potentially useful emerging visualization toolkits to provide a “living” arsenal for visualizing qualitative information in the meta-analytic evidence base.

## Data Availability

The data and analytical script to reproduce examples presented in this article are archived in GitHub repository at https://github.com/Yefeng0920/MA_Map_Bib.
